# PAH concentrations and exposure assessment from house dust retained in air-conditioning filters collected from Greater Doha, Qatar

**DOI:** 10.1007/s10653-019-00271-0

**Published:** 2019-03-27

**Authors:** Mohamed M. Mahfouz, Hassan M. Hassan, Elnaiem A. Elobaid, Oguz Yigiterhan, Balint Alfoldy

**Affiliations:** Environmental Science Center (ESC), Qatar University, H10-Zone 3-B113, P.O. Box: 2713, Doha, Qatar

**Keywords:** Indoor air quality, AC filter dust retained, BaP equivalent approach, PAH exposure assessment, Daily ingestion intake rate

## Abstract

Polycyclic aromatic hydrocarbons (PAHs) bound in dust retained in air-conditioning unit filters from 13 households in Greater Doha, Qatar, were quantified using GC–MS spectrometry. The median concentrations of ∑_16_PAH and ∑_7_PAH were 218.0 ng g^−1^ (± 125.3) and 112.1 ng g^−1^ (± 60.2) dry weight, respectively. Results show that except one sample, three- and four-benzene-ring PAHs were dominant in all dust samples. Phenanthrene, anthracene, pyrene, benzene(a)anthracene, and chrysene were dominant in 12 samples with maximum concentrations of 69.7 ng g^−1^ (± 24.0), 92.9 ng g^−1^ (± 28.1), 60.4 ng g^−1^ (± 14.7), 38.6 ng g^−1^ (± 7.3), and 14.7 ng g^−1^ (± 3.5), respectively. Benzo(k)fluoranthene has the most abundance of the quantified PAHs in the dust samples accounting for 19% of the total PAHs. Although Kriging interpolation shows a spatial variation of PAHs from north to south of Greater Doha, the mean concentrations in both directions were statically insignificant. Five samples displayed levels of benzo(a)pyrene (BaP) with maximum and median concentrations at 110.8 ng g^−1^ and 49.9 (± 28.4) dry weight, respectively. Benzo(a)pyrene equivalent approach $\left( {{\text{BaP}}_{\text{E}} } \right)$ was applied to assess carcinogenic exposure, and the resulting values (1.3–116.4 ng g^−1^) indicate that the levels observed were below the values reported for other countries within the region. Estimated daily ingestion (EDI) rates of PAHs retained in ACU filters were assessed for five age-groups < 1, 1–2, 3–6, 11–16, and > 19 years and were 0.39 (± 0.1), 0.33 (± 0.1), 0.20 (± 0.02), 0.07 (± 0.02), and 0.05 (± 0.01) ng kg^−1^/day, respectively. Source apportionment estimate indicates PAHs bound in dust retained in ACU filters are originated from pyrogenic sources.

## Introduction

Indoor particulate matter (PM) is a mixture of different materials that include street dust, suspended outdoor/indoor airborne dust which is usually bound with organic compounds such as polycyclic aromatic hydrocarbons (PAHs). PAHs are different carbon-based compounds comprised of fused aromatic rings arranged linearly, angularly, or in clusters (Ong et al. 2007). Their number is estimated to be in the hundreds, and they typically exist in the environment as complex mixtures, rather than individual compounds. Among the PAHs, 16 are classed as priority pollutants due to their health impact and carcinogenic potential. These include naphthalene (NAP), acenaphthene (ACE), fluorene (FLU), phenanthrene (PHE), anthracene (ANT), fluoranthene (FLN), pyrene (PYR), benzo(a)anthracene (BaA), chrysene (CHR), benzo(b)fluoranthene (BbF), benzo(k) fluoranthene (BkF), benzo(a) pyrene (BaP), indeno(1,2,3-cd)pyrene (IND), dibenzo(a,h)anthracene (DahA), and benzo(ghi)perylene (BghiP) (USEPA 2007). The last seven PAH compounds predominate in the particulate phase of atmospheric aerosols and are known to occur in the indoor dust.

Individuals inadvertently exposed to PAHs may develop respiratory damage (Whitehead et al. 2011a, b), where detrimental health effects depend on the level, nature, and duration of exposure (IARC 2010). Recent data suggest that PAH exposure increases the risk of leukemia and nervous system tumors in children, mainly if exposure occurs during the fetal stage of development and early childhood (Rengarajan et al. 2015a, b; Sánchez-Guerra and Quintanilla-Vegal 2013). Exposure pathways to indoor PAHs include (i) inhalation of cigarette smoke or smoke from open fireplaces, (ii) consumption of contaminated foodstuffs, ingestion of food and drink contaminated with deposited dust, and (iii) skin contact with contaminated surfaces.

Natural sources of air pollutants in Qatar can be significant due to the country’s arid climate, where the incidence of dust storms adversely affects air quality and enhances the air pollution. The Arabian Peninsula is also experiencing statistically significant climatic warming that reduces surface moisture and favors the mobilization of dust particulates (Alsarmi and Washington 2011; Zhang et al. 2005a). Moreover, recent studies show an escalating number of above-average warm days in the Arabian Peninsula, with an overall increase in extreme temperatures (Donat et al. 2013). In response, the residents of Qatar tend to spend most of their time indoors which thereby increases their exposure time to indoor air pollutants risks.

Most studies pertaining to this field assess indoor PAHs collected from floor/non-floor vacuumed dust and/or bulk dust samples (Gevao et al. 2007; Hoh et al. 2012; Maertens et al. 2008; Rengarajan et al. 2015a, b; Ong et al. 2007; Sánchez-Guerra and Quintanilla-Vegal 2013; Whitehead et al. 2011a, b), others evaluate the concentration of PAHs in indoor dust (Wang et al. 2017), with another group determining the concentration and source dynamics of PAHs for size-separated particles (Guinot et al. 2014). The current study evaluates PAHs extracted from indoor dust retained in filters housed inside air-conditioning units (ACUs). Although there are similar studies conducted within the region (Ali et al. 2016; Gevao et al. 2007), this study was the first in Qatar to identify probable sources and evaluate residents’ exposure via inhalation and ingestion of PAHs based on the application of toxicity factors and estimate daily intake rate of house dust.

## Materials and methods

Greater Doha represented by two main districts, Doha and Al Rayyan, where about 65% of Qatar’s population lives (MDPS Statistics 2016). It is surrounded by industrial cities 30 km to the north (Mesaieed industrial city), 60 km to the south (Ras Laffan) where the majority of Qatar’s oil and gas processing is located. Moreover, 12 km to the southwest, there is a dedicated industrial zone with large smelt workshops. The population census of the year 2015 showed that population density in the south of Greater Doha is higher than that in the north of the capital with 5000–10,000 person/km^2^ in the north and 10,000–39,662 person/km^2^ in the south (MDPS 2015) with higher traffic volumes, road capacities, building density, and number of occupants per building due to the dominance of the number of business companies.

**Fig. 1:**
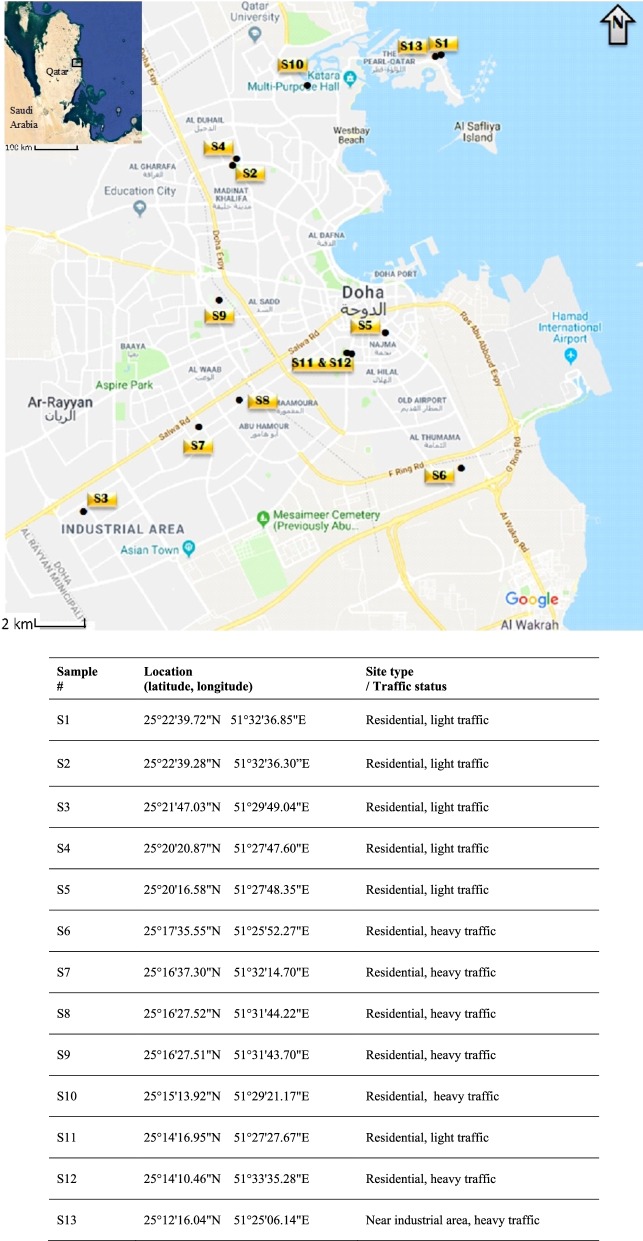
Samples collection locations, Greater Doha, Qatar (courtesy of Google Earth, 2018), and site and type of traffic within the location

After continued operations (3 months), 13 duplicate samples were collected from ACU filters representing different household indoor environments distributed geographically across the city. (All sampling locations were from medium-sized apartments and households, located in the ground and second floor of which the windows were rarely opened. All sampled ACU had the filters recently cleaned.) The sampling period was predefined to obtain maximum loadings of airborne dust during the summer months of 2015, i.e., May–September. Figure 1 displays the sampling locations. A 0.5–5.0 g sample of dust (dry weight) was collected from each ACU filter using a Teflon spatula pre-cleaned using n-hexane solvent. After collection, samples were placed in prewashed aluminum foil and stored at − 4.0 °C before analysis.

### Reagents and standards

Polyaromatic hydrocarbon analytical calibration standards were prepared from a certified reference standard, comprising of 16 PAH compounds at a concentration of 2000 µg/ml in dichloromethane (DCM) for each analyte (Restek^®^) and dissolved in DCM Sigma-Aldrich (Germany), using clean grade A glassware. The calibration range was from 5 to 500 ppb and utilized an internal standard containing five PAHs of ACE, CHR, NAP, PYR, and PHE at 200 µg/ml. Standards and samples were analyzed using an Agilent GC–MS system.

### Sample extraction and analysis

Samples dried in an oven set to 50 °C were extracted using a Dionex^®^ 200 accelerated solvent extractor (ASE). In essence, a 0.25-g sample is loaded into an ACE c.34 ml cell and backfilled with diatomaceous earth. The extract is then cleaned using SPE and concentrated to 1.0 ml under a gentle stream of pure nitrogen and a water bath at 40 °C and transferred into a 2.0-ml GC vial for analysis. The instrumental analysis was performed using a GC/MS fitted with a Restek Rxi^®^-5Sil MS, 30-m, 0.25-mm ID, 0.25-µm column and employed the following temperature program: 50 °C held for 30 s; ramped to 290 °C at 25 °C/min, and then to 320 °C at 5 °C/min. The flow rate was 23.5 ml/min.

### Quality assurance

Quality assurance was verified using the certified reference material (CRM) 1941b (*Organics in Marine Sediment*). Table 1 shows the recoveries of a selected PAHs analyte from the extracted CRM. The results show good analyte recovery ranging between 86 and 101%. Procedural blanks were prepared using the same analytical procedure and reagents but without an actual sample present. All sample analyses were in triplicate and performed at the Environmental Science Center (ESC) ISO 17025 accredited laboratories. The limit of detection was defined as three times the standard deviation of a blank reading. Detection limits varied between individual samples and PAHs depending on sample size and instrument sensitivity, respectively, and ranged from 1.1 (ANT) to 2.2 ng g^−1^ dry dust weight (NAP). PAHs found under the detection limit (< dl) have been considered by the concentration of (dl/√2) (Croghan and Egeghy 2005).

**Table 1:** Selected PAH recovery data from CRM 1941b

Analyte	Mean analyte result μg/kg (*n* = 2)	Certified value	% Recovery
Acenaphthene	33	38.4	86
Acenaphthylene	50	53.3	94
Benzo(b)fluoranthene	455.5	453	101
Chrysene	283	291	97
Naphthalene	775.5	848	91
Pyrene	383.5	397	97

### Statistical analysis

Descriptive statistical analysis of PAH data using a two-sample *t* test was performed using the Minitab statistical software version 17.0. Grubbs’ test (Grubbs 1969) to identify outliers in a univariate dataset assuming normal distribution. Contour maps of PAH concentrations were plotted using Surfer software.

### BaP equivalent concentration estimation (BaP_E_)

Benzo(a)pyrene (BaP) is the most toxic PAH identified toxicologically, and health risks from other PAHs are less characterized. In the present study, BaP equivalent concentration (${\text{BaP}}_{\text{E}} )$ was used to quantify the exposure. ${\text{BaP}}_{\text{E}}$ estimation was as per Cecinato (1997), Qi et al. (2014), Peng et al. (2012), and Yassaa et al. (2001) and defined as follows:

1 $${\text{BaP}}_{\text{E}} = 0.06 {\text{BaA}} + 0.07 {\text{B}}\left[ {b + k} \right]{\text{F}} + {\text{BaP}} + 0.6 {\text{DahA}} + 0.08 {\text{IND}}$$

where BaA, B[b + k]F, BaP, DahA, and IND are the individual concentrations of benzo(a)anthracene, benzo(b)fluoranthene + benzo(k)fluoranthene, benzo(a)pyrene, Dibenzo(a,h)anthracene, and Indeno(1,2,3-cd)pyrene, respectively.

### Estimated daily intake (EDI) of PAHs ingestion

Maertens et al. (2004) postulated that a dominant pathway by which chemicals enter the human body is via oral ingestion resulting from the hand-to-mouth transfer of contaminants (Maertens et al. 2004). EDI predicts the amount of dust that enters the human body via ingestion. In the present study, EDIs for PAHs retained in ACU filters were calculated by the following equation:

2 $${\text{EDI}} = C_{d} \cdot f/M$$

where $C_{d}$ represents the mean concentration of individual PAHs in the dust sample (µg g^−1^, dry weight); $f$ is the recommended dust ingestion limit value per day, i.e., 0.03 g/day for children less than 1 year and 0.06 g/day for others (USEPA 2008); and $M$ values are the mean body weight (kg).

## Results and discussion

The results of the mean concentration of each PAH along with the standard deviation (SD), variation coefficient, 95% confidence interval, and total concentrations of all 16 PAHs are displayed in Table 2. Outliers marked in bold were omitted from subsequent statistical analysis.

**Table 2:** PAH concentrations (ng g^−1^ dry weight) in indoor dust samples from Greater Doha, Qatar

Sample #	NAP	ACY	ACE	FLU	PHE	ANT	FLN	PYR	BaA	CHR	BbF	BkF	BaP	IND	DahA	BghiP	Σ_16_PAH	Σ_7_PAH
S1	< dl	< dl	< dl	0.7	14.7	17.4	3.6	24.1	35.6	5.2	44.1	78.1	< dl	< dl	< dl	< dl	223.5	163.0
S2	< dl	< dl	***25.5***	6.1	69.7	6.2	***175.5***	***79.5***	38.6	6	35.8	26.5	< dl	< dl	< dl	< dl	214.5	106.9
S3	5.7	0.1	< dl	0.2	2.5	54.8	1.4	16.4	23.5	4.4	21.6	45.5	49.9	8.1	5.3	< dl	239.4	158.3
S4	< dl	< dl	< dl	< dl	13.7	16.4	5.9	16.7	21.8	3.4	< dl	< dl	< dl	< dl	< dl	< dl	77.9	25.2
S5	5.0	0.1	< dl	0.1	0.4	9.1	< dl	4.9	10.6	1.5	20.8	41.3	< dl	3	0.5	< dl	97.3	77.7
S6	< dl	< dl	< dl	1.5	11.8	14.3	2.4	13.6	22.9	4	19.4	42.6	< dl	6.6	2.1	< dl	141.2	97.6
S7	1.0	0.2	< dl	0.4	< dl	< dl	< dl	< dl	< dl	< dl	< dl	< dl	< dl	< dl	< dl	< dl	1.6	0.0
S8	4.0	< dl	< dl	0.2	3.8	4.6	< dl	10.7	20.8	2.8	18.2	40.9	< dl	< dl	< dl	< dl	106.0	82.7
S9	22.3	0.1	< dl	1.7	38.4	46.4	18.6	22.3	20.9	14.7	31.6	48.3	64.1	6.9	6.7	< dl	343.0	193.2
S10	24.0	< dl	< dl	0.2	9.4	11.3	< dl	9.5	21	2.9	26.8	52.6	40.7	6.1	1.2	< dl	205.7	151.3
S11	< dl	2.0	1.1	< dl	62	51.9	< dl	24.4	23	5.6	***195.2***	28.2	46.4	6.8	2.1	< dl	251.5	112.1
S12	***52.0***	< dl	< dl	< dl	48.2	58.2	14.9	19	25.2	3.2	46.9	80.2	< dl	< dl	< dl	< dl	295.8	155.5
S13	16.6	< dl	2.7	5.9	17.5	92.9	50.3	60.4	29.2	***32.9***	18.3	35.7	110.8	***24.4***	***53.5***	***37.0***	477.3	194.0
Mean	11.2	0.1	9.8	1.7	24.3	32.0	13.9	20.2	24.4	4.9	28.4	47.3	62.4	6.3	3.0	–	206.0	117.0
SD	9.0	0.0	11.1	2.2	23.0	26.9	16.1	14.0	7.0	3.4	10.2	16.8	25.4	1.6	2.2	–	120.3	49.0
CV	0.9	0.4	1.1	1.3	1.0	0.8	1.2	0.7	0.3	0.7	0.4	0.4	0.4	0.3	0.8	–	0.6	0.4
CI	7.5	0.1	27.7	1.6	14.6	17.1	14.9	9.4	4.5	2.3	7.3	11.3	31.6	1.7	2.3	–	126.3	51.4

(<  dl) Lower than the detection limit, (*) data excluded due to low statistical significance. CI calculated for 95% confidence level. Outliers, marked in bold, determined by Grubb’s test omitted from statistical analysis

The results of the chemical analysis showed that PAHs with 2–3 aromatic rings (NAP, ACY, ACE, FLU, PHE, and ANT) have lower concentrations relative to 4–6 rings (FLN, PYR, BaA, CHR, BbF, BkF, BaP, IND, DahA, BghiP). The finding agrees with the properties of the molecule confirmed by Srogi (2007) who postulated that low molecular weight PAHs (2–3 rings) predominate in the vapor phase, whereas five- or more-ring PAHs tend to bind with particles and four-ring PAHs partition between vapor and particulates depending on the temperature (Srogi 007).

**Fig. 2:**
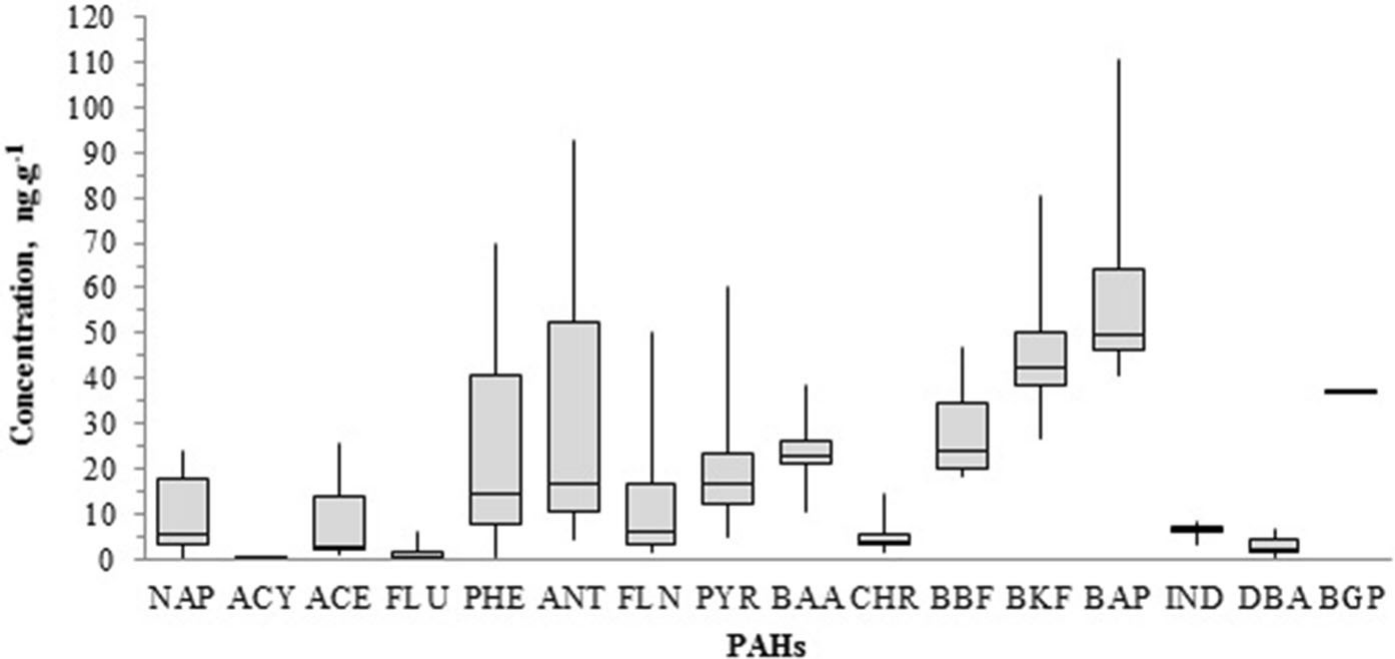
Median PAH concentrations with quartiles, minimum, and maximum concentrations

The total PAH concentrations (∑_16_PAHs) measured in the collected samples had a wide range of variation 1.6 ng g^−1^ (sample S7) to 477.3 ng g^−1^ (sample S13) with the mean value of 206.0 (± 125.3) ng g^−1^ (dry weight). Figure 2 shows the graphical representation of the median concentrations with quartiles, minimum and maximum values for individual PAH compounds.

Table 3 shows the mean concentration of individual PAH data published from research within similar atmospheric conditions and lifestyle in the region and worldwide. The table presents two type of samples: dust samples collected from the ACU filter and dust collected from surface deposed, since there is no statistically significant difference (*p* < 0.05) in mean concentrations for both types (Ali et al. 2016). Regionally, the mean of Σ_16_PAH in ACU filters in this study (0.2 µg.g^−1^) is an order of magnitude lower than values in Saudi Arabia (4.8 µg g^−1^), but similar to household floor dust in Kuwait (0.5 µg g^−1^) (Ali et al. 2016; Gevao et al. 2007). Other published data show higher concentrations from Italy (2.4 µg g^−1^) (Mannino and Orecchio 2008), Hong Kong (6.2 µg g^−1^) (Kang et al. 2010), Turkey (5.7 µg g^−1^) (Yilmaz Civan and Merve Kara 2016) and substantially elevated concentrations in house dust reported for Canada (29.0 µg g^−1^) (Maertens et al. 2008) and China (31.0 µg g^−1^) (Qi et al. 2014).

**Table 3:** Mean PAH concentrations (ng g^−1^) in indoor dust samples from nine different countries

	NAP	ACY	ACE	FLU	PHE	ANT	FLN	PYR	BaA	CHR	BbF	BkF	BaP	IND	DahA	BghiP	ΣPAH16	References
Qatar^a^	9.8	0.1	9.8	1.7	24.3	32.0	13.9	20.2	24.4	7.2	28.4	47.3	52.5	5.4	3.0	–	206	This study
Saudi Arabia (Jeddah)^a^	550	225	125	355	70	250	560	250	120	115	680	170	410	320	30	580	4800	Ali et al. (2016)
Saudi Arabia (Jeddah)^b^	175	110	160	290	105	425	425	385	90	95	700	110	550	15	15	105	3750	Ali et al. (2016)
Kuwait^b^	–	0.4	0.9	2.2	30.9	6.1	34.1	34.5	22.9	40.1	76.9	96.0	123.1	25.4	16.2	30.0	539	Gevao et al. (2007)
Italy (Palermo)^b^	–	1552.0	260.0	184.0	598.0	137.0	528.0	477.0	111.0	387.0	207.0	112.0	112.0	113.0	84.0	210.0	2426	Mannino and Orecchio (2008)
Hong Kong^b^	120.0	10.0	20.0	30.0	77.0	30.0	86.0	69.0	22.0	54.0	–	98.0	38.0	56.0	6.0	90.0	6180	Kang et al. (2010)
Australia^b^	124.2	6.9	201.7	12.6	8.8	3.6	53.6	77.3	6.4	5.4	11.3	3.5	3.0	3.3	20.8	6.1	–	Ong et al. (2007)
Turkey (Kocaeli)^b^	119.0	211.7	59.5	223.7	804.5	338.7	–	705.2	360.7	677.7	510.9	557.9	423.9	306.5	168.0	212.1	5680	Yilmaz Civan and Merve Kara (2016)
Canada (Ottawa)^**b**^	–	15	–	84	1530	222	–	1910	956	1460	2010	674	963	1290	250	1130	29,300	Maertens et al. (2008)
China^b^	849	173	93	464	4190	418	4260	2890	2370	1790	6230	763	1710	1970	510	2140	30,900	Qi et al. (2014)

^a^ACU filter sample

^b^Floor dust sample

(−) data unavailable

**Fig. 3:**
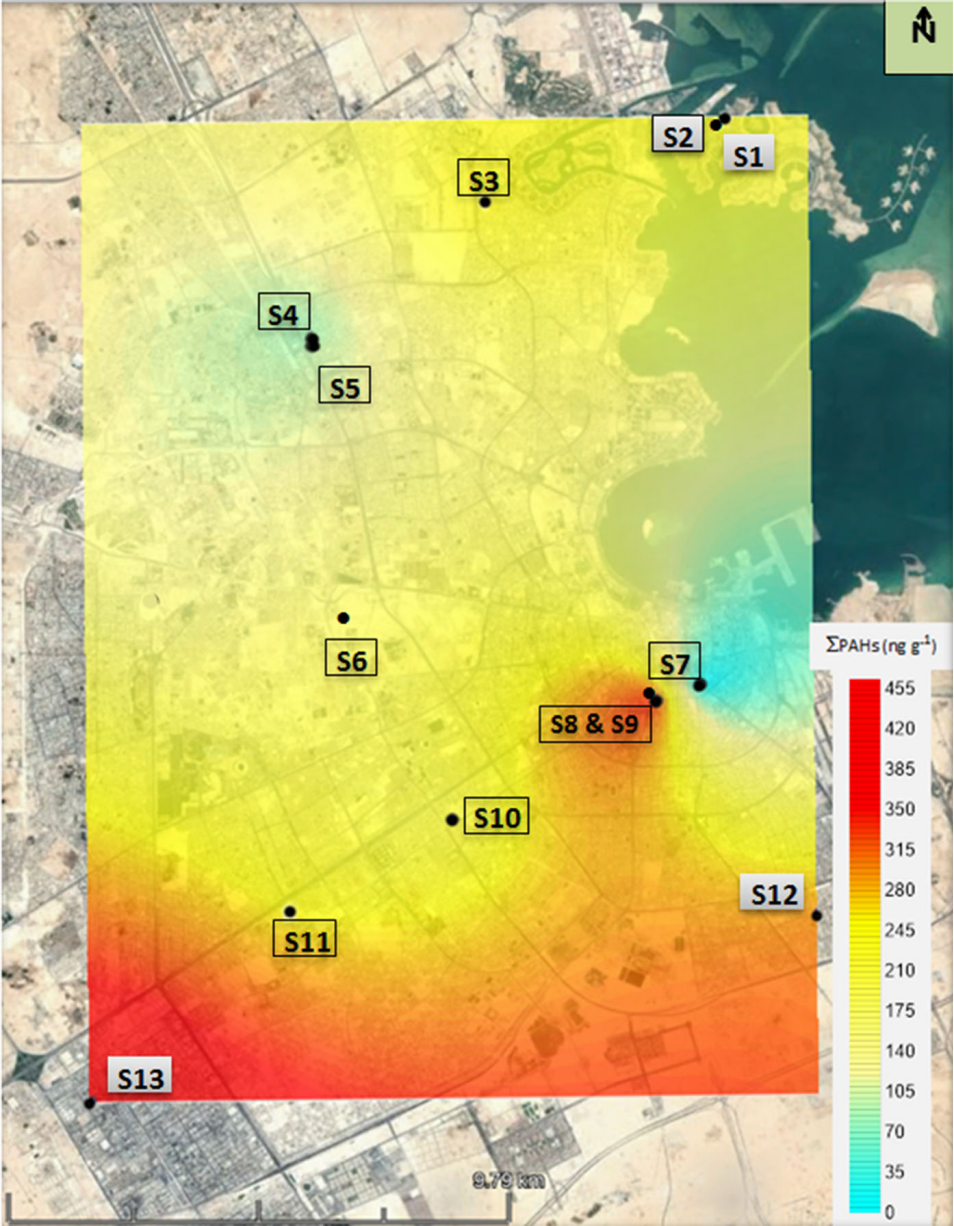
Kriging interpolation across Greater Doha of Σ_16_PAHs and (ng g^−1^)

Kriging interpolation of concentrations (Fig. 3) indicates heterogeneous variation within the PAHs in the dust samples from north to south in Greater Doha. PAHs concentration from outdoor sources influences their indoor levels. This variation may be linked to differences in traffic volume, population and buildings density being higher in the south than north of Greater Doha. The contours plot of ∑_16_PAHs concentrations shows elevated concentrations in the south of the city relative to the north. Mean individual PAH concentrations in the north and south of the city were statistically compared using two-sample t test, the results of which show that there was no significant difference in mean PAHs concentration between the north and south of Greater Doha. The apparent concentration gradient from north to south may be a consequence of elevated PAH concentrations detected in samples S13, S8, and S9.

The maximum concentration arising from the summation of the seven PAHs considered by the EPA and WHO to likely be carcinogenic was 194.0 ng g^−1^ detected in sample S13 (located in the south), while the mean and median of all samples were 117.0 and 112.1 (± 60.2) ng g^−1^, respectively.

Benzo(ghi)perylene, which has the highest molecular weight (278) of the PAHs, was detected in a single sample (S13), and this compound is typically formed via combustion at elevated temperatures (Steinhauer and Boehm 1992). Sampling point S13 is located adjacent to heavy traffic roads and local industry. Sample profiles also show that benzo(a)pyrene (BaP), the most carcinogenic PAH, was detected in only five out of the 13 samples and had an abundance of 12% with respect to all detected PAHs with an average concentration of 62.4 ng g^−1^ and a maximum concentration of 110.8 ng g^−1^ measured in sample S13. The photoreactivity of this compound can rationalize its limited abundance as postulated by Fraser et al. (1998) and Gogou et al. (1996). Under summer atmospheric conditions, PAHs will not transport far and are probably best used as local urban indicators.

Benzo(k)fluoranthene was the most abundant of the quantified PAHs in the ACU filters dust samples, accounting for 19% of the total PAHs. The three-benzene-ring PAHs (PHE, ANT), as well as four-benzene-ring PAHs (PYR, BaA, and CHR), were dominant in most ACU dust samples (*n* = 12 of 13 samples). Benzo(a) anthracene and chrysene are suspected to be human carcinogens (National Toxicology Program 2011), whereas the other PAHs (PHE, PYR, and ANT) are considered as pollutants. These two compounds typically present in coal, coke oven emissions, gasoline exhaust, and cigarette smoke (Hoh et al. 2012; Whitehead et al. 2011a, b).

### BaP_E_ calculations

Cities represent the highest risk factor associated with human exposure to airborne PAHs due to high population densities, traffic emissions, and restricted atmospheric dispersal of airborne PAHs. Inhalation of dust-containing PAHs is a key human exposure route. Many epidemiological studies found that exposure to PAHs may negatively affect pulmonary function in children and adults (Cakmak et al. 2017; Padula et al. 2015). The carcinogenic properties of PAHs, combined with their omnipresence in the urban atmosphere, represent a chronic health risk. Nevertheless, the quantification of PAHs exposure risk is a complex challenge (Tsai et al. 2002).

One approach to assess the exposure is using Eq. (1) to quantify ${\text{BAP}}_{\text{E}}$. The calculated ${\text{BaP}}_{\text{e}}$ values for indoor ACU filter dust samples in Greater Doha ranged from 1.3 ng g^−1^ (sample S4) to 116.4 ng g^−1^ (sample S13), with a mean of 33.4 (± 35.2) ng g^−1^. In comparison with similar studies in the region, the ${\text{BAP}}_{\text{E}}$ range in the present study is lower than that reported for ACUs filters in Saudi Arabia, i.e., 90–1000 ng g^−1^ with mean of 520 (± 335.0) ng g^−1^ (Ali et al. 2016) and mean of Kuwait floor dust samples, i.e., 160 ng g^−1^ (Gevao et al. 2007).

### Estimated daily intake (EDI)

Human exposure to soil pollutants in indirect way is usually considered a potentially important pathway. In order to define the EDI exposure risk of PAHs for residents of Greater Doha, the study data were evaluated according to age-groups, ranging from infants to adults in conjunction with exposure factors defined by the US EPA (US EPA 2011). Age-grouping allows computation of dust ingestion data using Eq. (2), where ingestion rates vary according to an individual’s age and body weight. Risk estimates represented in Table 4a show that the maximum EDI values for various age-groups, i.e., < 1, 1–2, 3–6, 11–16, and > 19 years old, were 0.39 (± 0.11), 0.33 (± 0.09), 0.20 (± 0.06), 0.07 (± 0.02), and 0.05 (± 0.01) ng kg^−1^/day, respectively. The EDI values for infants (< 1 year) reflect the highest exposure risk due to a higher incidence of hand-to-mouth behavior at this age. EDI-reported data worldwide are in general limited, and Table 4b shows a comparison of EDI for 16 PAHs for the adult age-group (> 19 years) in Qatar with EDIs from Saudi Arabia (Ali et al. 2016), Kuwait (Ali et al. 2016), China (Peng et al. 2012), Hong Kong (Kang et al. 2010), and the Netherlands (Oomen et al. 2008). Estimated EDIs in this study for PAHs exposure in Qatar are substantially less than the values reported for these other locations, with the highest EDI values reported for China.

**Table 4:** Estimated daily intake (EDI) rates for adult group (> 19 years) for Qatar and its comparison with international findings

Country	EDI (ng kg^−1^/day)																References
	NAP	ACY	ACE	FLU	PHE	ANT	FLN	PYR	BaA	CHR	BbF	BkF	BaP	IND	DahA	BghiP	
Qatar^a^	0.01	0.00	0.01	0.00	0.02	0.02	0.01	0.02	0.02	0.00	0.02	0.04	0.05	0.00	0.00	–	This study
The Netherlands^a^	0.20	0.01	0.40	0.40	0.30	0.10	0.10	0.30	0.20	0.30	–	0.40	0.20	0.20	0.10	1.70	Oomen et al. (2008)
Hong Kong^a^	0.10	0.01	0.02	0.02	0.61	0.02	0.68	0.55	0.17	0.43	–	0.78	0.30	0.46	0.05	0.70	Kang et al. (2010)
China^b^	1.27	0.13	0.05	0.18	1.17	0.08	0.95	0.70	0.24	0.56	–	0.66	0.37	0.40	0.08	0.47	Peng et al. (2012)
Kuwait^c^	0.19	0.06	0.05	0.16	0.02	0.02	0.12	0.03	0.06	0.04	0.17	0.05	0.10	0.04	–	0.05	Ali et al. (2016)
Saudi Arabia^c^	0.12	0.11	0.08	0.30	0.30	0.07	0.21	0.28	0.06	0.07	0.49	0.08	0.38	0.01	–	0.07	Ali et al. (2016)

^a^House

^b^Lecture theater

^c^House and car

(−) not available

### Source apportionment of PAHs

Source identification of PAHs contamination is an essential component of PAH characterization. PAH sources include petrogenic (petroleum sources), pyrogenic (derived from fuel and coal combustion or cigarette smoking) sources, and biogenic natural source(s). PAHs found in the indoor environment may have any of these sources (Saber et al. 2017). There are various methods of analyzing PAHs source apportionment, including abundance ratios of individual PAHs and the fossil fuel pollution index (Steinhauer and Boehm 1992). The ratios of PHE to ANT and FLN to PYR are one of the characteristics of origin (Okonkwo 1997). Chen et al. (2005), Yunker et al. (2002), and Zhang et al. (2005, b) have all proposed other ratios, such as the concentration ratio of anthracene (ANT) to the sum of ANT and PHE, and FLN to the sum of FLN and PYR (Chen et al. 2005; Yunker et al. 2002; Zhang et al. 2005, b). Brandli (2006) suggested that samples with an FLN:(FLN + PYR) ratio of less than 0.4 and an ANT:(ANT + PHE) ratio of less than 0.1 are of petrogenic origin, while those with an FLN/(FLN + PYR) ratio of less than 0.5 and an ANT/(ANT + PHE) ratio of greater than 0.1 are of pyrogenic origin. Moreover, samples with an FLN/(FLN + PYR) ratio of 0.4–0.5 may originate from fuel combustion, while ratios greater than 0.5 originate from grass, coal, and wood combustion (Brandli 2006).

Table 5 show the ratios of PAHs found in the ACU dust samples collected from Greater Doha. PAH ratios indicate that three of the samples (S13, S12, and S9) contain PAHs originating from a pyrogenic fuel combustion source(s) and vehicular emissions. These samples located in the south of the city were near heavily trafficked roads and local workshops. Two samples (S2 and S9) have biomass burning sources such as coal or wood burning nearby, and other samples (*n* = 8) contain PAH ratios indicating mixed pyrogenic origins attributed to the house occupants’ activities, i.e., cooking and usage of home incense in addition to vehicular emissions.

**Table 5:** Calculated FLN/(FLN + PYR) and ANT/(ANT + PHE) ratios for ACU dust in Greater Doha, Qatar

Sample #	FLN/(FLN + PYR)	ANT/(ANT + PHE)	Source apportionment
S1	0.13	0.54	Mixture pyrolysis
S2	0.16	0.96	Biomass burning
S3	0.45	0.84	Mixture pyrolysis
S4	0.26	0.54	Mixture pyrolysis
S5	0.54	0.43	Mixture pyrolysis
S6	0.44	0.55	Mixture pyrolysis
S7	0.04	0.46	Biomass burning
S8	0.09	0.55	Mixture pyrolysis
S9	0.15	0.55	Fuel combustion
S10	0.08	0.96	Mixture pyrolysis
S11	0.08	0.55	Mixture pyrolysis
S12	0.45	0.55	Fuel combustion
S13	0.54	0.08	Fuel combustion

## Conclusions

This study presents the preliminary levels for different PAHs bound in samples collected from house dust retained in air-conditioning filters. Sampled houses were geographically distributed in north and south of Greater Doha, Qatar. Results show that except one sample, three- and four-benzene-ring PAHs were dominant in all dust samples. Kriging interpolation of total PAHs concentration shows spatial variation from north to south, but statistical analyses did not confirm significant differences in mean PAH concentrations. Benzo (k) fluoranthene was the most abundant of the quantified PAHs accounting for 19% of the total concentration. Benzo(a)pyrene had the highest mean and maximum concentrations at 62.4 and 110.8 ng g^−1^, respectively. Compared to countries in the region, the concentrations of PAHs in indoor dust from Qatar are below those of Saudi Arabia and Kuwait. Human carcinogenic risk assessment of PAHs in ACU dust, based on the inhalation pathway, yielded a maximum ${\text{BAP}}_{\text{E}}$ value at 0.12 µg g^−1^, which is lower than that reported for similar studies in other countries within the region, i.e., Saudi Arabia (1.02 µg g^−1^) and Kuwait (0.16 µg g^−1^). Ingestion of dust in terms of estimates of maximum daily intake (EDI) for various age-groups (< 1, 1–2, 3–6, 11–16, and > 19 years old) was: 0.39 (± 0.11), 0.33 (± 0.09), 0.20 (± 0.06), 0.07 (± 0.02), and 0.05 (± 0.01) ng kg^−1^/day, respectively. The EDI values for the adult age-group (> 19 years) are substantially less than the values reported from other countries.

Analysis of isomers ratios of FLN:(FLN + PYR) and ANT:(ANT + PHE) shows that PAHs present in all indoor dust samples (*n* = 13) originate from a pyrolysis source(s) for which three samples show fuel combustion, two biomass burning, and eight mixture of pyrogenic source.
